# Model of care and chance of spontaneous vaginal birth: a prospective, multicenter matched-pair analysis from North Rhine-Westphalia

**DOI:** 10.1186/s12884-021-04323-1

**Published:** 2021-12-30

**Authors:** Sophia L. Tietjen, Marie-Therese Schmitz, Andrea Heep, Andreas Kocks, Lydia Gerzen, Matthias Schmid, Ulrich Gembruch, Waltraut M. Merz

**Affiliations:** 1 Department of Obstetrics and Prenatal Medicine, University Hospital Bonn, Venusberg-Campus 1, 53127 Bonn, Germany; 2 Department of Medical Biometry, Informatics and Epidemiology, Faculty of Medicine, University of Bonn, Venusberg-Campus 1, 53127 Bonn, Germany; 3 Directorate of Nursing, University Hospital Bonn, Venusberg-Campus 1, 53127 Bonn, Germany

**Keywords:** Pregnancy, Midwifery, Parturition, Obstetrics, Case-control studies, Prospective studies, Delivery rooms

## Abstract

**Background:**

Advantages of midwife-led models of care have been reported; these include a higher vaginal birth rate and less interventions. In Germany, 98.4% of women are giving birth in obstetrician-led units. We compared the outcome of birth planned in alongside midwifery units (AMU) with a matched group of low-risk women who gave birth in obstetrician-led units.

**Methods:**

A prospective, controlled, multicenter study was conducted. Six of seven AMUs in North Rhine-Westphalia participated. Healthy women with a singleton term cephalic pregnancy booking for birth in AMU were eligible. For each woman in the study group a control was chosen who would have been eligible for birth in AMU but was booking for obstetrician-led care; matching for parity was performed.

Mode of birth was chosen as primary outcome parameter. Secondary endpoints included a composite outcome of adverse outcome in the third stage and / or postpartum hemorrhage; higher-order obstetric lacerations; and for the neonate, a composite outcome (5-min Apgar < 7 and / or umbilical cord arterial pH < 7.10 and / or transfer to specialist neonatal care). Statistical analysis was by intention to treat. A non-inferiority analysis was performed.

**Results:**

Five hundred eighty-nine case-control pairs were recruited, final analysis was performed with 391 case-control pairs. Nulliparous women constituted 56.0% of cases. For the primary endpoint vaginal birth superiority was established for the study group (5.66%, 95%-CI 0.42% – 10.88%). For the composite newborn outcome (1.28%, 95%-CI -1.86% - -4.47%) and for higher-order obstetric lacerations (2.33%, 95%-CI -0.45% - 5.37%) non-inferiority was established. Non-inferiority was not present for the composite maternal outcome (-1.56%, 95%-CI -6.69% - 3.57%). The epidural anesthesia rate was lower (22.9% vs. 41.1%), and the length of hospital stay was shorter in the study group (*p* < 0.001 for both).

Transfer to obstetrician-led care occurred in 51.2% of cases, with a strong association to parity (p < 0.001). Request for regional anesthesia was the most common cause for transfer (47.1%).

**Conclusion:**

Our comparison between care in AMU and obstetrician-led care with respect to mode of birth and other outcomes confirmed the superiority of this model of care for low-risk women. This pertains to AMU where admission and transfer criteria are in place and adhered to.

## Introduction

Compared to obstetrician-led care, advantages of midwife-led models of care (MLC) have been reported, including an increased vaginal birth rate with less interventions, and a shorter duration of labor. Whereas obstetrician-led care is provided within hospital premises MLCs comprise a spectrum of places which include home (home births, HB), freestanding midwifery units (FMU), and alongside midwifery units (AMU). Midwife-led care may cover the entire period of pregnancy, birth and puerperium, or only parts of it, and may be organized as caseload, where one midwife (or a small group of midwives) is attending to a woman throughout pregnancy, birth and postpartum, and conventional type of care. Further variations in the spectrum of MLC arise from the fact that the provision of maternity service varies according to the health system, the status of midwives, and the extent of integration between the maternity care options, among others [1–7]. Research on this topic is therefore characterized by heterogeneity, which is further enhanced by differences in the study design, e.g. with respect to the choice of the control group, and the analysis according to intended or actual place of birth (for reviews see [8–11]).

In Germany, 98.4% of women are giving birth in obstetrician-led units, where interprofessional service is provided [12]. The units are classified in four levels according to equipment and expertise, with level Four being a basic obstetrician-led delivery unit without neonatal services or expertise in the provision of care for pregnant women with complications and level One a perinatal unit for pregnancies complicated by very early preterm birth and maternal or fetal abnormalities, and intensive care units for both, the mother and the newborn. The remaining 1.6% of births are cared for exclusively by midwives, predominantly in FMUs and at home.

Alongside midwifery units have been established in Germany in 2003 [13]. AMUs are hospital-based and located within the same premises. Here, midwife-led care is offered for low-risk women; continuous, one-to-one care is being provided. Criteria for admission to AMU and transfer to obstetrician-led care before, during and immediately after labor are in place; they have been jointly agreed upon by the respective team of midwives and obstetricians. Women are attended to by the same team of midwives who also care for women giving birth in the obstetrician-led model. Even though these births are entered into the national birth registry, they are not specifically labelled [14], thus precluding a nation-wide survey.

The aim of our study was to compare the maternal and perinatal outcome of births taking place in AMUs in North Rhine-Westphalia (NRW), Germany’s most populous federal state, with the outcome of carefully selected low-risk women giving birth in obstetrician-led care at the same unit. Additionally intervention rates, transfer rates and causes, and length of hospital stay were analyzed.

## Methods

A prospective, controlled, multicenter study was conducted. All AMUs situated in NRW were invited to participate. The AMUs were localized in obstetric units of all levels of care. Six of the seven AMUs in NRW participated. The study was approved by the Ethics Committee of the University Bonn Medical School (registration number 254/18). Recruitment took place from November 2018 to September 2020.

All pregnant women booking for birth at the AMU of one of the six study sites were eligible. A checklist, jointly developed by the respective team of obstetricians and midwives, was in place at all study sites. Here, inclusion and exclusion criteria for birth in AMU as well as indications for transfer to obstetrician-led care before, during, and immediately after labor were specified. Each study site applied its own checklist; however, all checklists were based on a blueprint developed by a group of researchers in midwifery [13] with local adaptations [15].

Inclusion criteria were healthy women with a singleton, term (≥37 weeks of gestation), cephalic pregnancy.

Exclusion criteria consisted of specific features in the medical or obstetric history, e.g. preexisting medical conditions or previous cesarean section (CS); complications during pregnancy, e.g. preeclampsia; and fetal features like small or large for gestational age or oligohydramnios.

Indications for transfer from AMU to obstetrician-led care during birth included delayed first or second stage of labor; need for augmentation of labor; fetal heart rate abnormalities; pyrexia or other maternal adverse events arising during labor; request for regional or i.v.-opioid analgesia. In the immediate postpartum period hemorrhage, higher-degree obstetric lacerations, and other complications of the third stage of labor (e.g. incomplete placenta) were indications for transfer to obstetrician-led care.

The control group was chosen as follows: for each woman booking for birth at the respective AMU the subsequent woman booking for obstetrician-led care at the same unit was recruited. Women in the control group fulfilled exactly the same inclusion and exclusion criteria. Additionally, matching for parity was performed (nulliparous and parous).

Women were informed about the study during their booking visit at the respective birthing unit by the attending midwife or physician and invited to participate. All participants gave their informed consent.

### Sample size calculation

Power and sample size calculations were based on the retrospective analysis of births in the AMU of the coordinating study site [16]. For the primary endpoint ‘mode of birth’, the required number of cases was determined using a non-inferiority test for paired binary endpoints [17]. Assuming a rate of cesarean/instrumental vaginal births of 10% with a difference of 2.5% between groups (discordant cases) and a non-inferiority margin of 2% as in the retrospective analysis, 692 cases are needed to achieve a power of 80% with a level of 5% (two-sided, normal approximation).

The following data were collected:

For the parturient:

Parity; mode of birth; epidural analgesia; labor duration; episiotomy; obstetric lacerations; adverse events during third stage including postpartum hemorrhage; admission-to-discharge time. For women who were transferred to obstetrician-led care, time and cause of transfer was noted.

For the newborn:

Birth weight; birth weight percentile; Apgar score after 1, 5 and 10 min; umbilical artery (UA) pH; UA Base Excess (BE), unplanned transfer to specialist neonatal care.

The following outcomes were defined with regard to the non-inferiority analysis: The primary endpoint ‘mode of birth’ covered the rate of cesarean/instrumental vaginal births. The secondary endpoints included a composite outcome of 5′-Apgar score < 7 and / or UA-pH < 7.10 and / or unplanned transfer to specialist neonatal care; a composite outcome of adverse events during third stage including postpartum hemorrhage; and third- or fourth-degree perineal or cervical (i.e. higher-order obstetric) lacerations.

### Statistical methods

Statistical analysis was by intention to treat. Comparisons were performed according to the intended model of care during birth. Basic characteristics of the study and control groups were summarized using descriptive statistics, with numbers (with percentages) and mean values (with standard deviations) reported for valid cases only. Differences between both groups were evaluated using McNemar’s tests for categorical variables and paired sample t tests or Wilcoxon signed-rank tests for continuous variables. A non-inferiority analysis was performed for the primary and all secondary outcomes. The difference between paired proportions of each outcome together with the 95% confidence interval (CI) was determined. Non-inferiority of AMUs to obstetrician-led care was declared if the lower bound of the confidence interval of the difference did not fall below the non-inferiority margin of 2%. For the study group, characteristics of a transfer to obstetrician-led care during or immediately after birth were summarized and differences according to parity were evaluated using Chi Square tests. The relation between transfer and length of hospital stay was evaluated using Wilcoxon tests. Further, a linear mixed-effects model with adjustments for study site was used to analyze the association between duration of birth (dependent variable) and parity and transfer (both as independent variables). Analyses were carried out using R (version 4.0.2) and SAS® Software (version 9.4, SAS Institute Inc. Cary, NC, USA).

## Results

**Fig. 1:**
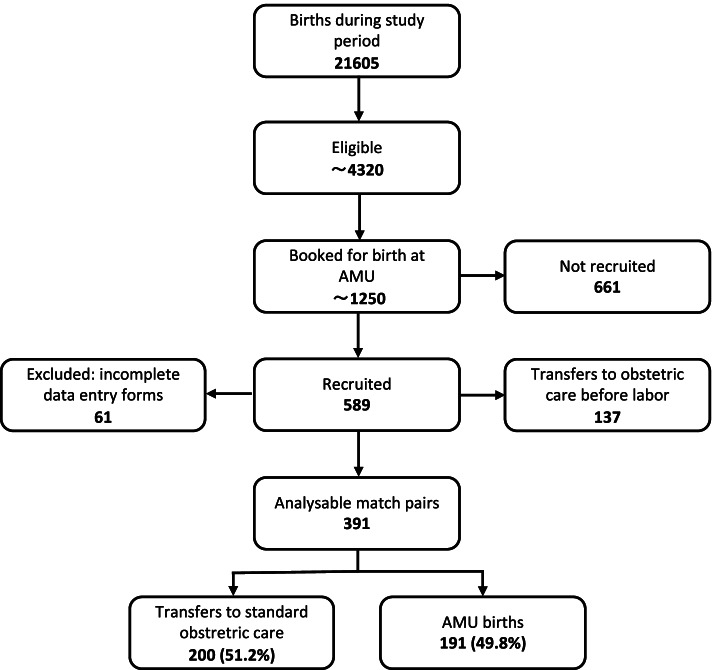
Recruitment Flow

An overview of the recruitment process is depicted in Fig. 1. Overall, 21,605 women gave birth during the study period in the participating sites. Of these, 589 women planning birth in AMU (corresponding to 2.7% of all women, and 13.6% of those assumed to be eligible) [15] were recruited. Incomplete data entry forms of women who decided to give birth at another institution reduced the number of study and control pairs available for analysis to 528 (89.6%). Between recruitment and admission to labor ward, 137 (25.9%) women were transferred to obstetrician-led care, 90 (65.7%) of them for medical reasons; these included induction of labor for preterm rupture of membranes, post-date pregnancy, or suspected fetal growth restriction, and other maternal or fetal specific features like preeclampsia or prematurity.

The final analysis was therefore performed with the group of women (*n* = 391) who were admitted for labor in AMU in one of the six study sites, i.e. who intended to give birth in AMU, and their matched controls (*n* = 391). In six cases (1.1%) matching was incorrectly performed: nulliparous women were matched with parous women in two cases, and four times vice versa. These cases were included in the analysis.

Table 1 summarizes maternal, obstetric, and neonatal data of the study and control group. The majority of participants were nulliparous (*n* = 219, 56.0%). The spontaneous vaginal birth rate was higher in the study group (82.8% vs. 77.0%), the request for epidural analgesia was lower (22.9% vs. 41.1%). Birth with intact perineum occurred more often in the study group (30.1% vs. 22.5%). No difference was observed in the episiotomy rate between the study and control group. The postpartum hemorrhage rate, defined as blood loss > 500 ml during vaginal birth (> 1000 ml for CS, respectively) was higher in the study group (13.5% vs. 11.5%).

**Table 1:** Characteristics of the study and control group (*n*=391)

	Study group (*n* = 391)		Control group (*n* = 391)		*p* value
Maternal characteristics of non-inferiority analysis					
Nulliparous (n, %)	219	56.0	216	55.2	-
Mode of birth (n, %)					-
Spontaneous	322	82.8	301	77.0	
Instrumental vaginal	39	10.0	38	9.7	
Cesarean	28	7.2	52	13.3	
Missing	2		0		
Obstetric injury (n, %)					-
First degree perineal laceration, labia laceration	75	21.3	75	22.8	
Second degree perineal laceration, vaginal or clitoral laceration	165	46.9	168	51.1	
Third or fourth degree perineal laceration, cervical laceration	6	1.7	12	3.6	
None	106	30.1	74	22.5	
Missing*	39		62		
Adverse outcome in the third stage (n, %)	30	7.7	31	8.0	-
Missing	2		4		
Postpartum hemorrhage (n, %)	52	13.5	44	11.5	-
Missing	6		8		
Further maternal characteristics					
Duration of labor (min), (mean, SD)	433.6	334.9	443.9	317.0	0.663^#^
Missing	6		9		
Episiotomy (n, %)	35	9.8	31	9.3	0.888
Missing^a^	33		56		
Epidural anesthesia (n, %)	89	22.9	158	41.1	<0.001
Missing	2		7		
Hospital discharge within six hours after birth (n, %)	88	23.1	27	7.0	<0.001
Missing	10		5		
Length of hospital stay (days), admission to discharge (mean, SD)	2.8	1.2	3.3	1.2	<0.001
Missing^b^	104		42		
Length of hospital stay (days), birth to discharge (mean, SD)	2.3	1.0	2.7	1.0	<0.001
Missing^b^	105		42		
Neonatal characteristics of non-inferiority analysis					
5-minute APGAR score < 7 (n, %)	2	0.5	5	1.3	-
Missing	1		1		
Umbilical cord arterial pH < 7.10 (n, %)	7	1.8	9	2.3	-
Missing	6		4		
Unplanned transfer to specialist neonatal care (n, %)	10	2.6	15	3.9	-
Missing	6		5		
Further neonatal characteristics					
Birthweight (gram), mean (SD)	3475.2	391.3	3461.9	399.7	0.664
Missing	1		3		
Birthweight percentile, mean (SD)	49.7	25.5	47.1	26.7	0.173
Missing	91		89		
1-minute APGAR score (mean, SD)	8.9	0.7	8.8	1.0	0.305
Missing	1		2		
5-minute APGAR score (mean, SD)	9.8	0.6	9.7	0.8	0.855
Missing	1		1		
10-minute APGAR score (mean, SD)	10.0	0.2	9.9	0.3	0.035
Missing	1		1		
Umbilical cord arterial pH (mean, SD)	7.3	0.1	7.3	0.1	0.080
Missing	6		4		
Umbilical cord arterial Base Excess (mean, SD)	-5.6	3.4	-5.4	3.4	0.163
Missing	10		9		

*SD* standard deviation

^#^adjusted for study site

^a^including cases with mode of birth=cesarean

^b^including women who left the hospital for home six hours after birth

We found no statistically significant difference in birth weight, birth weight percentile, UA pH and BE, nor in 1′-, 5′-, and 10′-Apgar scores between the groups. Nearly one quarter of women in the study group (23.1%) decided to leave the hospital for home 6 hours after birth compared to 7.0% in the control group. For those who stayed as in-patients, the mean duration of their hospital stay (admission to discharge: 2.8 vs. 3.3 days (SD 1.2), and birth to discharge: 2.3 vs. 2.7 days (SD 1.0)) was shorter.

Table 2 contains the non-inferiority analysis for the predefined primary and secondary outcomes. For mode of birth, the analysis revealed superiority for the study group (5.66%, 95%-CI 0.42% – 10.88%). Non-inferiority was established for the newborn composite outcome (1.28%, 95%-CI -1.86% – 4.47% and for higher-order obstetric lacerations 2.33%, 95%-CI -0.45% – 5.37%). Non-inferiority, however, could not be demonstrated for the composite maternal outcome (-1.56%, 95%-CI -6.69% – 3.57%).

**Table 2:** Non-inferiority analysis (*n*=391)

	n^a^	Study group	Control group	Difference (%)	95%- CI	*p* value
Cesarean/ instrumental vaginal birth	389	67	89	5.66	(0.42 – 10.88)	0.002
Neonatal composite outcome^+^	390	17	22	1.28	(-1.86 – 4.47)	0.021
Maternal composite outcome^++^	385	65	59	-1.56	(-6.69 –3.57)	0.452
Higher-order obstetric injury^+++^	300	5	12	2.33	(-0.45 – 5.37)	0.001

^+^Umbilical cord arterial pH < 7.10 and/or 5-minute APGAR < 7 and/or unplanned transfer to specialist neonatal care

^++^Adverse outcome in the third stage and/or postpartum hemorrhage

^+++^Third or fourth degree perineal laceration or cervical laceration; CI = confidence interval.

^a^Number of pairs for non-inferiority analysis differs from total (*n*=391) due to missing values.

Transfer rates, time, and causes are listed in Table 3. Two hundred women (51.2%) were transferred from AMU to obstetrician-led care; in the majority of cases, transfer took place during labor (87.5%, *n* = 175). There was a strong association between parity and transfer (*p* < 0.001). Nulliparous women constituted the majority of transfers (76.5%, *n* = 153), and request for epidural analgesia was the most common cause (47.1%). For parous women, the leading cause for transfer was fetal heart rate abnormalities (63.9%). Compared to their matched controls there was no difference in the obstetric and neonatal outcome for women after transfer except for a higher postpartum hemorrhage rate (*n* = 200, 26.0% vs. 12.2%, *p* < 0.001, data not shown).

**Table 3:** Study group: Transfer times, rates and causes to standard obstetric care according to parity (*n*=391)

	Total (*n*=391)		Nulliparous (*n* = 219)		Parous (*n* = 172)		*p* value
Transfer to obstetric care (n, %)	200	51.2	153	69.9	47	27.3	<0.001
In case of transfer: transfer time (n, %)							<0.001
During labor	175	87.5	139	90.8	36	76.6	
After birth	25	12.5	14	9.2	11	23.4	
Missing^a^	191		66		125		
In case of transfer during labor: transfer causes, categorized (n, %)							
Fetal	59	33.7	36	25.9	23	63.9	<0.001
Obstetric	32	18.3	29	20.9	3	8.3	
Non-medical causes	84	48.0	74	53.2	10	27.8	
Missing^b^	216		80		136		

^a^including no transfer

^b^including transfer after birth or no transfer

**Fig. 2:**
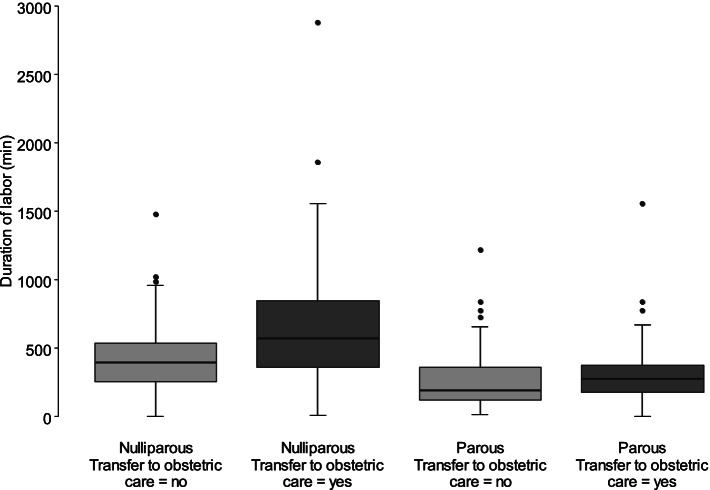
Study group: Duration of labor according to parity and transfer

**Fig. 3:**
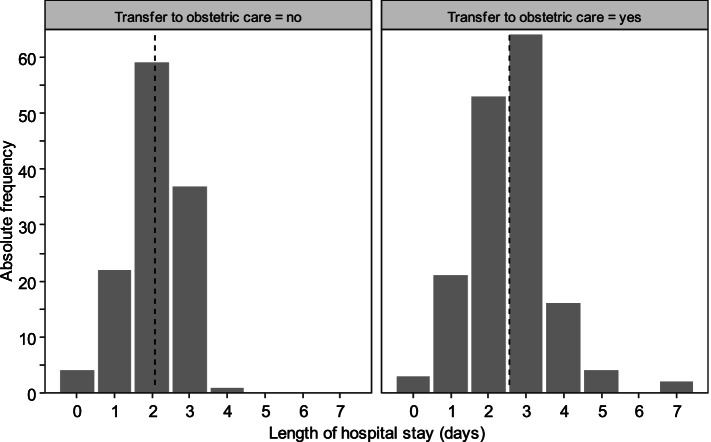
Study group: Length of hospital stay (from birth to discharge) according to transfer. Dashed line: mean

Overall, the duration of labor did not differ between the study and control group. For the study group, regression analysis revealed an association between parity, transfer to obstetrician-led care, and duration of labor (see Fig. 2). Likewise, the length of the hospital stay (from birth to discharge) was longer in women who were transferred from AMU to obstetrician-led care (2.6 vs. 2.1, *p* < 0.001, see Fig. 3).

## Discussion

Our analysis of midwife-led care in alongside midwifery units in NRW revealed superiority of this model of care with respect to our primary endpoint mode of birth (spontaneous vaginal versus instrumental vaginal and CS). Since the recruitment took place at all but one AMUs in NRW, the control group consisted of matched, low-risk women, and an intention-to-treat protocol was applied we are confident our result is valid.

This result is in line with our previous single-center retrospective investigation; there, we found a trend towards higher spontaneous and lower instrumental vaginal births [16]. The comparison with other studies is difficult for various reasons; these pertain to differences in the study design, the choice of the control group, and variations in the provision of maternity care, among others. Three studies on this topic were restricted to low-risk women expecting an uncomplicated labor. Bernitz et al. 2011 in their randomized single-center study used operative birth as primary endpoint to examine three models of care (midwife-led unit, normal, and special unit, all located within one hospital); no difference was found [18]. McLachlan et al. 2012 performed a randomized study and compared caseload with standard obstetric care. The spontaneous vaginal birth rate was higher in the former model (RR 1.13, 95%-CI 1.06–1.21) [2]. The “Birthplace in England Study” analyzed the perinatal outcome according to intended place of birth; compared to obstetric units the adjusted odds ratio for spontaneous vaginal birth was higher in all models of care under investigation [19]. Other investigations and reviews compared home birth or birth in FMUs with hospital birth [3–7, 11].

Three systematic reviews investigated the association between various models of care, including AMU, and obstetric outcomes. Bohren et al. 2017 analyzed continuous support during childbirth versus standard care and found a positive effect on the spontaneous vaginal birth rate (RR 1.08, 95%-CI 1.04–1.12). Continuous support was not limited to midwives but included any labor companion [9]. Sandall et al. 2016 in their meta-analysis compared midwife-led models of care - comprising continuity of care during pregnancy, birth and postpartum – with other models of care and found higher spontaneous vaginal birth rates (RR 1.05, 95%-CI 1.03–1.07). Pregnant women of low and high risk were included in the analysis [8]. Scarf et al. 2018 restricted their analysis to high-income countries. Here, the odds ratio for spontaneous vaginal birth was higher in birth centers (encompassing AMUs and FMUs) compared to planned hospital births (estimated OR 1.92, 95%-CI 1.59–2.32). The majority of included studies were retrospective; additionally, parity was not accounted for [10].

The non-inferiority analysis of our secondary endpoints revealed varied results: For higher-order obstetric lacerations non-inferiority could be established. In our previous investigation the rate of higher-order obstetric lacerations was higher in the study group [16]. A higher mean birthweight and a higher number of newborns with birthweight > 4500 g in the study group may have contributed to this outcome. Other studies and reviews reported a similar trend to our present study: Bernitz et al. 2011 and McLachlan et al. 2012 did not find a difference in the rate of higher-order obstetric lacerations [2, 18]. The “Birthplace in England study” revealed a lower adjusted odds ratio of third- and fourth-degree perineal tears for multiparous women giving birth at home or in FMU [19]. The systematic reviews by Sandall et al. 2016 and Bohren et al. 2017 did not differentiate the extent of the perineal laceration [8, 9]. No difference was found with respect to severe perineal trauma in the systematic review by Scarf et al. 2018 [10].

The perinatal outcome of births in midwife-led models of care has been thoroughly investigated. We defined a composite outcome since severe perinatal morbidity or mortality is a rare event [16]. Here, we did not include umbilical artery base excess into the analysis since it is not routinely reported in the international literature. Our study was underpowered for this research question. Nevertheless, the confirmation of non-inferiority in AMU with respect to the perinatal outcome is reassuring. Bernitz et al. 2011 did not find a difference in the perinatal outcome parameters under investigation [18]. A lower admission rate to special-care nursery with no difference in the admission rates to the neonatal intensive care unit (NICU) was reported by McLachlan et al. 2012 [2]. A composite primary perinatal outcome was chosen by the “Birthplace in England” authors. Here, for all but nulliparous women giving birth at home (adjusted odds ratio 1.75, 95% CI 1.07–2.86) the primary outcome occurred significantly less often in midwife-led models of care [1]. The systematic reviews revealed similar results: no difference was present for selected perinatal outcome parameters (perinatal mortality > 24 weeks of gestation plus neonatal mortality; 5′-Apgar score < 7; neonatal convulsions; NICU admission) in the systematic review comparing midwife-led continuity models versus other models of care [8]. A lower rate of low 5′-Apgar score (RR 0.62; 95% CI 0.46–0.85) was reported in births after continuous support in the systematic review by Bohren et al. 2017. For other selected perinatal outcome parameters (NICU admission, prolonged neonatal hospital stay) no difference was detected [9]. Scarf et al. 2018 examined perinatal mortality and NICU admission; no difference was present for these outcome parameters between the different models of care [10].

We could not confirm non-inferiority for our secondary composite endpoint maternal outcome, comprising adverse events during the third stage of labor including postpartum hemorrhage. This result is in contrast to our retrospective analysis. There, no difference was present in the postpartum hemorrhage rate between study and control group [16]. Likewise, above mentioned studies and reviews did not find a difference in the maternal outcome [2, 8, 18, 19]. The result of our composite maternal outcome analysis was mainly determined by a higher postpartum hemorrhage rate in the study group. An explanation for our result may be the overall high postpartum hemorrhage rate (13.5% in the study group, 11.5% in the control group, corresponding to a difference of eight cases). Postpartum hemorrhage was defined as blood loss > 500 ml after vaginal birth (1000 ml after CS, respectively). The diagnosis did not require any quantitative confirmation. We assume that midwives at the study sites had an overall low threshold to diagnose postpartum hemorrhage. Another explanation for our finding may be the eschewal of routine prophylactic oxytocin administration in AMU. This intervention is known to reduce blood loss > 500 and > 1000 ml after vaginal birth [20]. Further studies on this topic should preferably apply quantitative methods for measurement of blood loss.

We additionally compared selected interventions. We found a significantly lower epidural analgesia rate in the study group. This result is in line with all published studies [2, 8, 9, 18, 19]. There was no difference in the episiotomy rate, which was low in both, study and control group (9.8 and 9.3%, respectively). Episiotomy rates vary greatly between countries and healthcare systems, and even within countries and models of care [21, 22].

With respect to hospital stay our results indicated that women in the study group favored early hospital discharge. McLachlan et al. 2012 in their study found a reduction in the length of postpartum hospital stay (55.4 h, SD 0.97 versus 60.5 h, SD 0.78, *p* < 0.001) in their caseload group [2]. No difference in postpartum hospital stay was detected in the systematic review by Sandall et al. 2016 [8]. Our findings may indicate that women opting for care in AMU may have different values with respect to their childbirth and aim for an experience without interventions and minimal contact time with a hospital environment.

More than half (51.2%) of the parturients were transferred to obstetrician-led care. Nulliparous women constituted the majority of transfers, and request for epidural analgesia was the most common cause. In Germany, midwife-led models of care do not allow for interventions like oxytocin augmentation or administration of i.v.-opioid analgesia. These factors may have contributed to the high transfer rate. Additionally, the fact that obstetrician-led care is available within the same premises; possible without delay; and with continuing care of the parturient by the respective midwife before and after transfer may have lowered the threshold for a decision in favour of transfer.

An explanation for our finding that transfer rates were dominated by nulliparous women requesting analgesia may be owed to the fact that during the study period a severe shortage of hospital-based midwives was present. Pregnant women were anxious about the quality of care they would receive for their labor. Since care in AMU included continuous one-to-one care, women may have registered for birth in AMU with the major intention to get high-quality care for their birth, and less with the aim to give birth without interventions. Our results confirm our previous retrospective data; here, the transfer rate was 50.3% [16]. Transfer rates and causes from midwife-led models of care to obstetrician-led care have been extensively examined; in the majority of reports transfers pertain to hospital from home. For all investigations, an association between parity and transfer rate was established. Here, additional information may support women’s informed choice with respect to model of care for birth. A German single-center analysis reported 14.6% transfers from home to hospital [23]. Transfers were separately analyzed for the “Birthplace in England” study. Here, transfer from AMU occurred in 27.0% (21.0% from FMUs, respectively), with an adjusted odds ratio for transfer from AMU of 2.6 for nulliparous women. Prolonged labor was the most common cause (35%) [24]. Blix et al. 2014 in their systematic review reported overall transfer rates between 9.9 and 31.9% (nulliparous: 23.4–45.4%; parous 5.8–12.0%) [25]. In a study from Oregon / USA 16.5% of women were transferred from home to hospital during labor [4]. In a New Zealand study, transfer from FMUs occurred in 53.1%; these included transfers before the onset of birth [5, 26]. In a study from Denmark, transfer rates of 28.4% from home to hospital were reported [6]. Seijmonsbergen-Schermers et al. 2020 described a transfer rate of 55–68% for nulliparous women in the Netherlands (20–32% for parous women, respectively) [21].

Strengths of our study include the prospective design; the conduction in obstetric departments of all levels of care; the meticulous selection of cases and controls – only women entering labor after uneventful pregnancy with a high chance for an uncomplicated vaginal birth were recruited; the analysis according to the intended place of birth; the predefined transfer criteria; and the reporting of the outcome of transferred cases.

Limitations of our study include the size of our study group. The reduction in recruitment was mainly owed to the Covid pandemic which forced all participating study sites to change the booking procedures for birth. This included either a switch from personal to electronic booking or abandoning booking procedures for low-risk women altogether, thereby reducing the chances of recruitment. Additionally, as a result of shortage of staff and rising number of births, midwives of all study sites reported a very high workload during the study period. These time constraints resulted in women not being invited to participate despite their eligibility and was the rationale for choosing a one-to-one ratio for the recruitment of study and control group even though a one-to-three ratio would have increased the statistical power of our study.

## Conclusion

In summary, our comparison between care in AMU and obstetrician-led care with respect to mode of birth and other outcomes confirmed the superiority of this model of care for low-risk women, and add important information to the ongoing discussion about this model of care. Our findings however are only valid if criteria for admission to AMU and transfer to obstetrician-led care are defined and adhered to.

## Data Availability

The datasets used and/or analysed during the current study are available from the corresponding author on reasonable request.

## Abbreviations

AMU: Alongside midwifery unit
BE: Base excess
CI: Confidence interval
CS: Cesarean section
FMU: Freestanding midwifery unit
HB: Home birth
i.v.: Intravenous
MLC: Midwife-led care
NICU: Neonatal intensive care unit
NRW: North Rhine-Westphalia
SD: Standard deviation
UA: Umbilical artery
